# Preoperative treatment with 5α-reductase inhibitors and the risk of hemorrhagic events in patients undergoing transurethral resection of the prostate – A population-based cohort study

**DOI:** 10.6061/clinics/2018/e264

**Published:** 2018-03-07

**Authors:** Ti-Yuan Yang, Marcelo Chen, Wun-Rong Lin, Chung-Yi Li, Wei-Kung Tsai, Allen W. Chiu, Ming-Chung Ko

**Affiliations:** I Department of Urology, Mackay Memorial Hospital, Taipei, Taiwan; II Department of Medicine, Mackay Medical College, Taipei, Taiwan; III Department of Cosmetic Applications and Management, Mackay Junior College of Medicine, Nursing and Management, Taipei, Taiwan; IV Department of Public Health College of Medicine, National Cheng Kung University, Tainan, Taiwan; V Department of Public Health, College of Public Health, China Medical University, Taichung, Taiwan; VI School of Medicine, National Yang-Ming University, Taipei, Taiwan; VII Department of Urology, Taipei City Hospital, Taipei, Taiwan; VIII Department of Health Care Management, National Taipei University of Nursing and Health Sciences, Taipei, Taiwan

**Keywords:** Transurethral Resection of the Prostate, Benign Prostatic Hyperplasia, 5-alpha-reductase Inhibitors, Blood Transfusion

## Abstract

**OBJECTIVES:**

To assess the associations between preoperative treatment with 5-alpha reductase inhibitors and the risks of blood transfusion during transurethral resection of the prostate and blood clot evacuation or emergency department visits for hematuria within 1 month after surgery.

**METHODS:**

We used data from the Taiwan National Health Insurance Research Database in this population-based cohort study. A total of 3,126 patients who underwent first-time transurethral resection of the prostate from 2004 to 2013 were identified. Adjusted odds ratios estimated by multiple logistic regression models were used to assess the independent effects of the preoperative use of 5-alpha reductase inhibitors on the risks of perioperative hemorrhagic events after adjustment for potential confounders.

**RESULTS:**

Two hundred and ninety-seven (9.4%) patients were treated with 5-alpha reductase inhibitors for <3 months, and 65 (2.1%) patients were treated for ≥3 months prior to undergoing transurethral resection of the prostate. The blood transfusion rates for patients who were not treated with 5-alpha reductase inhibitors (controls), patients who were treated with 5-alpha reductase inhibitors for <3 months, and patients who were treated with 5-alpha reductase inhibitors ≥3 months were 9.5%, 8.8%, and 3.1%, respectively. 5-alpha reductase inhibitors tended to decrease the risk of blood transfusion; however, this association was not statistically significant (adjusted odds ratio=0.14, 95% confidence interval: 0.02-1.01). Age ≥80 years, coagulopathy, and a resected prostate tissue weight >50 g were associated with significantly higher risks of blood transfusion than other parameters.

**CONCLUSIONS:**

This nationwide study did not show that significant associations exist between 5-alpha reductase inhibitor use before transurethral resection of the prostate and the risks of blood transfusion and blood clot evacuation or emergency visits for hematuria.

## INTRODUCTION

Transurethral resection of the prostate (TURP) is the current standard operative procedure for the management of bothersome lower urinary tract symptoms caused by benign prostatic hyperplasia (BPH) 1. Perioperative hemorrhagic events are common TURP-related complications 2. The incidence rate of bleeding requiring transfusion reportedly ranges from 0.4% to 7.1% 3. In addition to necessitating blood transfusion, perioperative hemorrhage may also result in blood clot retention, which may require surgical intervention 4.

5-alpha-reductase inhibitors (5ARIs), such as finasteride and dutasteride, reduce prostate volume by suppressing dihydrotestosterone synthesis 5. Hagerty et al. 6 first reported that pretreatment with finasteride seemed to be useful in reducing the incidence of perioperative bleeding and, consequently, the need for return visits to the emergency department and transfusions. This beneficial effect may be attributable to the fact that finasteride causes decreases in vascular endothelial growth factor (VEGF) expression and inhibits angiogenesis, leading to decreases in microvessel density (MVD) in prostatic suburethral tissues 7. Additionally, Kravchick et al. 8 reported that dutasteride may exert similar effects to reduce the vascularity of the prostate. However, other studies have not reported that the preoperative use of 5ARIs provides significant benefits with respect to the prevention of hemorrhagic events in patients undergoing TURP 9-11. Thus, the aim of this nationwide population-based study was to determine the association between preoperative treatment with 5ARIs and the risk of perioperative hemorrhagic events.

## MATERIALS AND METHODS

### Study design and data source

This population-based cohort study used data from the Taiwan National Health Insurance Research Database (NHIRD). The national health insurance (NHI) program in Taiwan is a single-payer payment system that was implemented in March 1995 and currently covers almost 99.5% of the entire population of Taiwan 12. The NHIRD is provided by the National Health Research Institutes (NHRI) in Taiwan for research purposes and contains “cohort datasets” including the claims data for 1 million people randomly sampled from all beneficiaries in 2000, 2005, and 2010. The purpose of these cohort datasets is to provide researchers with groups representative of the population that can be followed longitudinally 13. In this study, we used the longitudinal health insurance dataset 2000 (LHID2000), which contains the medical claims data for 1 million subjects randomly sampled from all beneficiaries in 2000. According to the NHRI, there is no significant difference in gender between the group of patients in the LHID2000 and that in the original NHIRD 14.

### Study participants and 5ARI prescriptions

Subjects who underwent TURP for BPH from 2004 to 2013 were identified using the procedure codes for TURP (International Classification of Diseases, 9th Revision, Clinical Modification (ICD-9-CM) codes: 79406B, 79411B, 79412B, 79413B, 79414B, and 79415B) and the ICD-9-CM diagnosis codes (600.x) for BPH. If a patient underwent TURP more than once from 2004-2013, only the first procedure was included in the analysis. Patients diagnosed with prostate cancer (ICD-9-CM code: 185) prior to undergoing TURP were excluded from the study.

Information on 5ARI (finasteride or dutasteride) prescriptions was retrieved from both inpatient and outpatient claims filed during a 6-month period prior to TURP. We divided the patients who did and did not receive preoperative treatment with 5ARIs into the following three categories a category comprising patients who did not receive any 5ARIs within 6 months before TURP (controls), a category comprising patients who were prescribed 5ARIs for <3 months in the 6-month period prior to TURP, and a category comprising patients who were prescribed 5ARIs for ≥3 months in the 6-month period prior to TURP.

### Study outcomes

The study outcomes were blood transfusion at the time of TURP and blood clot evacuation or emergency department visits for hematuria within 1 month following TURP.

To identify patients who underwent blood transfusions at the time of TURP, we used the medical order codes related to blood transfusion, including those related to the fees for blood transfusion, blood products, cross-matching tests, and antibody screening. Only study subjects with claims data for all of the above medical order codes were considered to have received blood transfusions. Information regarding blood clot evacuation was obtained with a procedure code (50030C) for bladder blood clot evacuation, and information on emergency department visits was obtained using the codes for emergency physician fees and the diagnostic code for hematuria (ICD-9-CM code: 599.7).

### Covariates

Age at enrollment, co-morbidities, and medical institutions were considered covariates in this study. Information on co-morbidities, such as hypertension (ICD-9-CM code: 401-405), diabetes (ICD-9-CM code: 250), coagulopathy (vitamin K deficiency (ICD-9-CM code: 269.0), coagulation defects (ICD-9-CM code: 286), thrombocytopenia (ICD-9-CM codes: 287.3, 287.4, 287.5, 287.8, and 287.9), liver failure (ICD-9 CM codes: 5712, 5715, and 5716), and end-stage renal disease (ICD-9-CM code: 585), was obtained with the appropriate diagnostic codes. Co-morbidities were counted only in cases in which a subject had been hospitalized at least once or completed three or more outpatient visits for specific diagnoses within 1 year prior to TURP. Additionally, study subjects who received aspirin, warfarin, or clopidogrel for ≥3 months within 6 months prior to TURP were considered to have used anticoagulants. Subjects whose records included the diagnostic code for urinary retention (ICD-9-CM code: 788.20) and who received a medical order for indwelling urinary catheterization at the same outpatient visit, emergency department visit, or hospitalization within the 6-month period before TURP were considered to have experienced urinary retention. Moreover, the surgeries were classified into <15-g, 15-50-g, and >50-g groups based on the weight of the resected prostate tissue and the amounts for which patients were reimbursed. The accreditation of the medical institutions (i.e., medical centers, regional hospitals, and district hospitals) at which TURP was performed was also considered a covariate in the analysis. Medical institutions were included in the study because of possible dissimilarities in clinical practices among urologists and operation outcomes.

### Statistical analysis

We first described the baseline covariates for the study subjects according to the different degrees to which the subjects had been exposed to 5ARIs. We then calculated the rates of blood transfusion and blood clot evacuation or emergency department visits for hematuria for the three study groups. We also performed multiple logistic regression to assess the independent effect of preoperative treatment with 5ARIs on the risks of the perioperative hemorrhagic events of interest after adjusting for covariates. All statistical analyses were performed using Statistical Analysis Software, version 9.4 (SAS Institute Inc., Cary, NC, USA). A *p*-value <0.05 was considered statistically significant.

### Ethics

The Institutional Review Board of National Cheng Kung University Hospital (IRB #B-ER-102-120-t) reviewed and approved the study protocol and granted access to the NHIRD.

## RESULTS

Of the 3,126 patients who underwent TURP, 297 (9.4%) received preoperative treatment with 5ARIs for <3 months, and 65 (2.1%) received treatment for ≥3 months. Those who received 5ARI treatment for ≥3 months tended to be younger and to have higher prevalences of hypertension and urinary retention but lower prevalences of diabetes and coagulopathy than other patients. Those patients also had higher TURP resection volumes and underwent TURP in medical centers or regional hospitals more frequently than other patients (Table 1).

Table 2 shows the rates of blood transfusion according to the different levels of exposure to 5ARIs. The overall blood transfusion rate was 9.3%, and the blood transfusion rate was 9.5% in patients treated without 5ARIs, 8.8% in patients treated with finasteride or dutasteride for <3 months and 3.1% in patients treated with finasteride or dutasteride for ≥3 months. The odds ratios were 0.90 (95% CI, 0.57-1.43, *p*=0.6651) and 0.14 (95% CI, 0.02-1.01, *p*=0.0506) after adjustment for baseline characteristics. The adjusted odds ratio (AOR) for blood transfusion was significantly elevated in patients aged ≥80 years compared to patients aged 70-79 years (1.65; 95% CI, 1.21-2.25, *p*=0.0017). Additionally, diabetes (AOR, 1.39, 95% CI, 1.00-1.92, *p*=0.0486) and coagulopathy (AOR, 1.69; 95% CI, 1.21-2.37, *p*=0.0022) were associated with a significantly increased risk of blood transfusion. Patients with a resected prostate tissue weight >50 g had a significantly higher risk of blood transfusion than patients with a resected prostate tissue weight <15 g (AOR, 3.77; 95% CI, 2.56-5.56, *p*<0.0001). Patients who underwent TURP at regional hospitals also had a significantly higher risk of blood transfusion than patients who underwent surgery at other medical centers (AOR, 1.49, 95% CI, 1.11-1.99). The blood transfusion rates for patients treated without 5ARIs (controls), patients treated with 5ARIs for <3 months, and patients treated with 5ARIs for ≥3 months were 15.7%, 10.9%, and 0%, respectively, in patients with coagulopathy. The blood transfusion rates for patients treated without 5ARIs (controls), patients treated with 5ARIs for <3 months, and patients treated with 5ARIs for ≥3 months were 8.6%, 8.4%, and 3.9%, respectively, in patients without coagulopathy. The multiple logistic regression model suggested that the differences in the blood transfusion rates between the two groups were not significant.

The overall rate of blood clot evacuation or emergency department visits for hematuria within 1 month after TURP was 2.5%, which was similar to the corresponding rates for patients treated without 5ARIs (2.6%), patients treated with 5ARIs for <3 months (2.0%), and patients treated with 5ARIs for ≥3 months (3.1%). The corresponding rates for patients aged <60, 60-69, 70-79, and ≥80 years were 3.1%, 2.6%, 3.0%, and 3.4%, respectively. The multiple logistic regression model also suggested that there was no significant association between preoperative treatment with 5ARIs and the risk of blood clot evacuation or emergency department visits for hematuria within 1 month after TURP. The only factors that were significantly associated with an increased risk of blood clot evacuation or an emergency department visit for hematuria were a resected prostate tissue weight >50 g (AOR: 3.46, 95% CI, 1.63-7.34) and operations at district hospitals (AOR: 2.24, 95% CI, 1.11-4.52) (Table 3).

## DISCUSSION

Whether or not pretreatment with 5ARIs decreases bleeding during TURP remains controversial 5-11. The mechanism underlying the association between 5ARIs and decreases in prostatic bleeding may involve decreases in VEGF expression and angiogenesis inhibition. Pareek et al. 7 provided histochemical insight into the mechanism by which finasteride reduces MVD in prostatic sub-urethral tissue over a minimum of 6 weeks before surgery. Kravchick et al. 8 used trans-rectal color Doppler sonography to demonstrate that prostate tissue vascular density was reduced after 6 weeks of treatment with dutasteride. Additionally, Busetto et al. 15 concluded that pretreatment with dutasteride for 8 weeks reduced VEGF indices, MVDs and bipolar TURP bleeding rates in patients with large prostates with a volume ≥50舁ml.

A previous meta-analysis of eight randomized controlled trials including 565 cases was conducted to evaluate the need for blood transfusion for TURP and revealed that the effect of treatment with 5ARIs on the need for transfusion was favorable. However, the results showed that the difference in the need for blood transfusion did not differ significantly between the treatment and control groups 16. Interpreting the findings of this meta-analysis was difficult because of dissimilarities in patient characteristics resulting from study-to-study differences in exclusion criteria. For example, Sandfeldt et al. 17 excluded patients with coagulation disorders and those with a large prostate volume (>90 ml), while Ozdal et al. 18 excluded patients who received aspirin, warfarin or similar anticoagulant drugs prior to surgery, and Donohue et al. 19 excluded subjects with a creatinine concentration greater than 150 μmol/L and subjects on aspirin. Hahn et al. 11 excluded patients with liver disease, bleeding disorders, and a history of anticoagulant drug use, abnormal liver function test results, unstable cardiovascular disease, acute or chronic renal failure, or hematological disease. Kravchick et al. 8 excluded patients with infections, acute urinary retention and indwelling catheters, and Pastore et al. 20 excluded patients with coagulation disorders. Because the prevalences of coagulopathy, anticoagulant use and urinary retention are high in patients with BPH, the exclusion of these co-morbidities may have greatly limited the generalizability of the results of above studies.

To the best of our knowledge, our study is the largest population-based work to investigate the effect of pretreatment with 5ARIs on the risk of TURP-related hemorrhagic events. Our results showed that preoperative 5ARIs use did not significantly decrease the risks of blood transfusion or blood clot evacuation or emergency department visits after TURP. Pretreatment with 5ARIs reduced blood loss 19; however, it is possible that the reduction in blood loss was not sufficient to result in a measurable decrease in transfusion rates. The factors associated with the retention of blood clots needing evacuation and emergency department visits for hematuria after TURP may be complex, as they may be associated not only with TURP but also with postoperative care. In addition, patient access to medical institutions may also affect the frequency of emergency department visits. Patients with a TURP resection weight greater than 50 g also had a significantly higher rate of blood clot evacuation or emergency department visits for hematuria within 1 month post-surgery than patients with lower TURP resection weights. The associations between resected prostate tissue weight and surgical complications, including surgical re-intervention and TURP-related blood transfusion, have been reported previously 4.

Our study found that patients aged ≥80 years, those with a resected prostate tissue weight ≥50 g and those with coagulopathy had a higher risk of blood transfusion than other patients. Elderly patients have more chronic diseases and comorbidities, reduced body function, and more complex diseases and display poorer adherence to therapy regimens, which may increase their risk of a blood transfusion 21. An association between resected prostate tissue weight and TURP-related blood transfusion was also reported in a previous study 4. Resected prostate tissue weight may be correlated with larger prostatic urethral wound surface areas and higher risks of bleeding. Coagulopathy is a known predictor of massive bleeding in patients undergoing liver transplantation 22. However, coagulopathy has not previously been identified as a predictor of perioperative hemorrhagic events in patients undergoing TURP. Our study noted a positive association between coagulopathy and the risk of blood transfusion, an association that may be attributable to the fact that coagulopathy increases the difficulty of intraoperative hemostasis, causes blurring of the operative field, and causes more intraoperative bleeding.

The use of anticoagulants preoperatively was not associated with significantly higher rates of perioperative hemorhagic events in our study. Chen et al. 23 reported that in Taiwan, most surgeons order their patients to stop taking anticoagulants at 7 days before TURP to avoid increasing the risk of surgical bleeding. Our study examined the effect of treatment with anticoagulants over a 6-month period prior to surgery, which may explain the null association between anticoagulant use and perioperative hemorrhagic events.

Our study found that patients who underwent TURP in regional and district hospitals had a significantly higher risk of perioperative hemorrhagic events than those treated in medical centers. It seems that previous studies have not specifically examined the influence of medical institution accreditation on TURP outcomes, including perioperative hemorrhagic events; however, lessons can be learned from existing studies on volume-outcome relationships in surgery and from advanced sophisticated medical examinations. Our data showed that 33.3% of TURPs were performed in medical centers. Based on our data and given the limited number (*n*=19) of medical centers in Taiwan, it is likely that the case volume at the medical centers was high. Our finding that higher TURP case volumes led to better outcomes with respect to perioperative hemorrhagic event rates is in line with those of previous studies on the outcomes of surgical treatments 24,25 and advanced medical examinations, such as an endoscopic retrograde cholangiopancreatography 26. Whether the hypothesis of “practices makes perfect” also hold trues for TURP warrants further investigation.

Only 362 (11.6%) patients were prescribed 5ARIs prior to TURP in our study. This small percentage may be due to the regulation of Taiwan's NHI program, which allows the prescription of 5ARIs only if the maximum urinary flow rate is <15 ml per second, or the prostate volume is >30 ml as measured by transrectal ultrasound. Physicians other than urologists may not be familiar with these examinations and therefore may not treat their patients with 5ARIs when required. However, long-term 5ARI use has been reported to reduce the risks of acute urinary retention and BPH-related surgery; therefore, fewer patients on 5ARIs need surgery 27,28. Taken together, these findings may explain why we were able to identify only a small percentage of patients who were prescribed 5ARIs before surgery.

There were some limitations to this study. Certain data relevant to perioperative hemorrhagic events, namely, data pertaining to preoperative and postoperative hemoglobin levels, platelet counts, prothrombin times, and activated partial thromboplastin times and data pertaining to operative times, were not available in the NHIRD. The unavailability of these data may have resulted in residual confounding. Additionally, since this was a retrospective study, it was not possible to confirm if the medications were actually used by the patients. Non-adherence to prescribed medication regimens may have influenced the study results. However, this study also had the following strengths: first, the use of insurance claims data for clinical research provided us with easy access to the longitudinal records for a large sample of geographically dispersed patients, which made it possible for us to perform analyses according to certain variables of interest, including age, associated comorbidities, and medications. Second, this was the first study to evaluate perioperative hemorrhagic events in patients undergoing TURP and to include coagulopathy data in the analysis. Third, the data used in this study were retrieved from the medical claims data from the NHIRD; thus, the likelihood of recall bias, nonresponses, or loss to follow-up was small.

In conclusion, this nationwide study did not demonstrate that a significant association exists between 5ARI use before TURP and the risks of blood transfusion and blood clot evacuation or emergency visits for hematuria. Patients older than 80 years, patients with TURP resection weights greater than 50 g and patients with a history of coagulopathy have a significantly higher risk of blood transfusion after TURP than other patients. A TURP resection weight greater than 50 g is associated with a significantly higher rate of blood clot evacuation or emergency department visits for hematuria within 1 month after TURP than a lower TURP resection weight.

## AUTHOR CONTRIBUTIONS

Yang TY and Chen M were responsible for the manuscript writing and editing. Lin WR, Tsai WK and Chiu AW were responsible for the protocol and project development. Li CY was responsible for the data collection, management and analysis. Ko MC was responsible for the manuscript writing and editing, and protocol/project development.

## Figures and Tables

**Table 1:** Baseline characteristics of patients with different durations of exposure to 5-alpha-reductase inhibitors.

Characteristics		5-alpha-reductase inhibitor prescriptions		
	Total (%)	None n (%)	<3 months n (%)	≥3 months n (%)
	3126 (100.0)	2764 (88.4)	297 (9.4)	65 (2.1)
Age (years)				
<60	256 (8.2)	226 (8.2)	25 (8.4)	5 (7.7)
60-69	922 (29.5)	809 (29.3)	91 (30.6)	22 (33.8)
70-79	1322 (42.3)	1174 (42.5)	119 (40.1)	29 (44.6)
≥80	626 (20.0)	555 (20.1)	62 (20.9)	9 (13.8)
Hypertension				
No	1768 (56.6)	1572 (56.9)	153 (51.5)	43 (66.2)
Yes	1358 (43.4)	1192 (43.1)	144 (48.5)	22 (33.8)
Diabetes				
No	2557 (81.8)	2262 (81.8)	246 (82.8)	49 (75.4)
Yes	569 (18.2)	502 (18.2)	51 (17.2)	16 (24.6)
Coagulopathy				
No	2722 (87.1)	2420 (87.6)	251 (84.5)	51 (78.5)
Yes	404 (12.9)	344 (12.4)	46 (15.5)	14 (21.5)
Anticoagulant				
No	3007 (96.2)	2661 (96.3)	283 (95.3)	63 (96.9)
Yes	119 (3.8)	103 (3.7)	14 (4.7)	2 (3.1)
Urinary retention				
No	2410 (77.1)	2140 (77.4)	214 (72.1)	56 (86.2)
Yes	716 (22.9)	624 (22.6)	83 (27.9)	9 (13.8)
TURP volume				
5∼15 g	1357 (43.4)	1198 (43.3)	134 (45.1)	25 (38.5)
15∼50 g	1504 (48.1)	1329 (48.1)	139 (46.8)	36 (55.4)
>50 g	265 (8.5)	237 (8.6)	24 (8.1)	4 (6.2)
Hospital accreditation*				
Medical center	1042 (33.3)	900 (32.6)	115 (38.7)	27 (41.5)
Regional hospital	1283 (41.0)	1132 (41.0)	122 (41.1)	29 (44.6)
District hospital	477 (15.3)	454 (16.4)	17 (5.7)	6 (9.2)

TURP: transurethral resection of the prostate.

* The inconsistencies between the total population and the individual populations were caused by missing information.

**Table 2:** Rates of and odds ratios for blood transfusion according to the different durations of exposure to 5-alpha-reductase inhibitors and various baseline characteristics.

	No. of people with blood transfusion	Adjusted odds ratio for blood transfusion event*		
	n (%)	Estimates	(95% CI)	*p*-value
Overall	291 (9.3)			
Exposure to 5ARI				
None	263 (9.5)	1.00		
<3 months	26 (8.8)	0.90	(0.57-1.43)	0.6651
≥3 months	2 (3.1)	0.14	(0.02-1.01)	0.0506
Age (years)				
<60	9 (3.5)	0.36	(0.16-0.78)	0.0098
60-69	65 (7.1)	0.82	(0.59-1.13)	0.2279
70-79	124 (9.4)	1.00		
≥80	93 (14.9)	1.65	(1.21-2.25)	0.0017
Hypertension				
No	153 (8.7)	1.00		
Yes	138 (10.2)	1.05	(0.80-1.37)	0.7210
Diabetes				
No	226 (8.8)	1.00		
Yes	65 (11.4)	1.39	(1.00-1.92)	0.0486
Coagulopathy				
No	232 (8.5)	1.00		
Yes	59 (14.6)	1.69	(1.21-2.37)	0.0022
Anticoagulant				
No	278 (9.3)	1.00		
Yes	13 (10.9)	0.91	(0.47-1.78)	0.7887
Urinary retention				
No	209 (8.7)	1.00		
Yes	82 (11.5)	1.24	(0.92-1.66)	0.1543
TURP volume				
5∼15 g	93 (6.9)	1.00		
15∼50 g	134 (8.9)	1.26	(0.94-1.69)	0.1249
>50 g	64 (24.2)	3.77	(2.56-5.56)	<0.0001
Hospital accreditation*				
Medical center	83 (8.0)	1.00		
Regional hospital	142 (11.1)	1.49	(1.11-1.99)	0.0075
District hospital	38 (8.0)	1.04	(0.69-1.57)	0.8619

TURP: transurethral resection of the prostate.

* Adjusted odds ratios were estimated from the multiple logistic regression model, in which all the variables listed in Table 2 were included as independent variables.

**Table 3:** Rates of and odds ratios for blood clot evacuation or emergency department visits for hematuria according to the different durations of exposure to 5-alpha-reductase inhibitors and various baseline characteristics.

	No. of people with blood clot evacuation or emergency department visits for hematuria	Adjusted odds ratio for blood clot evacuation or emergency department visits for hematuria		
	n (%)	Estimates	(95% CI)	*p*-value
Overall	79 (2.5)			
Exposure to 5ARI				
None	71 (2.6)	1.00		
<3 months	6 (2.0)	0.54	(0.17-1.74)	0.2995
≥3 months	2 (3.1)	1.49	(0.35-6.34)	0.5881
Age (years)				
<60	8 (3.1)	1.82	(0.76-4.32)	0.1774
60-69	24 (2.6)	1.04	(0.55-1.96)	0.9021
70-79	26 (2.0)	1.00		
≥80	21 (3.4)	1.38	(0.72-2.63)	0.3343
Hypertension				
No	40 (2.3)	1.00		
Yes	39 (2.9)	0.96	(0.57-1.63)	0.8931
Diabetes				
No	65 (2.5)	1.00		
Yes	14 (2.5)	1.09	(0.58-2.08)	0.7827
Coagulopathy				
No	65 (2.4)	1.00		
Yes	14 (3.5)	1.22	(0.60-2.45)	0.5817
Anticoagulant				
No	74 (2.5)	1.00		
Yes	5 (4.2)	2.14	(0.82-5.61)	0.5817
Urinary retention				
No	60 (2.5)	1.00		
Yes	19 (2.7)	1.13	(0.63-2.00)	0.6852
TURP volume				
5∼15 g	27 (2.0)	1.00		
15∼50 g	37 (2.5)	1.53	(0.86-2.72)	0.1486
>50 g	15 (5.7)	3.46	(1.63-7.34)	0.0013
Hospital accreditation*				
Medical center	17 (1.6)	1.00		
Regional hospital	31 (2.4)	1.48	(0.81-2.70)	0.2030
District hospital	16 (3.4)	2.24	(1.11-4.52)	0.0246

TURP: transurethral resection of the prostate.

* Adjusted odds ratios were estimated from the multiple logistic regression model, in which all the variables listed in Table 2 were included as independent variables.
